# Protective Role of Polyphenols against Vascular Inflammation, Aging and Cardiovascular Disease

**DOI:** 10.3390/nu11010053

**Published:** 2018-12-28

**Authors:** Alexa Serino, Gloria Salazar

**Affiliations:** 1 Department of Nutrition, Food and Exercise Sciences, Florida State University, Tallahassee, FL 32306, USA; als15e@my.fsu.edu; 2 Center for Advancing Exercise and Nutrition Research on Aging (CAENRA), Florida State University, Tallahassee, FL 32306, USA

**Keywords:** NADPH oxidases, polyphenols, senescence, VSMCs, ECs, ROS, cardiovascular disease

## Abstract

Aging is a major risk factor in the development of chronic diseases affecting various tissues including the cardiovascular system, muscle and bones. Age-related diseases are a consequence of the accumulation of cellular damage and reduced activity of protective stress response pathways leading to low-grade systemic inflammation and oxidative stress. Both inflammation and oxidative stress are major contributors to cellular senescence, a process in which cells stop proliferating and become dysfunctional by secreting inflammatory molecules, reactive oxygen species (ROS) and extracellular matrix components that cause inflammation and senescence in the surrounding tissue. This process is known as the senescence associated secretory phenotype (SASP). Thus, accumulation of senescent cells over time promotes the development of age-related diseases, in part through the SASP. Polyphenols, rich in fruits and vegetables, possess antioxidant and anti-inflammatory activities associated with protective effects against major chronic diseases, such as cardiovascular disease (CVD). In this review, we discuss molecular mechanisms by which polyphenols improve anti-oxidant capacity, mitochondrial function and autophagy, while reducing oxidative stress, inflammation and cellular senescence in vascular smooth muscle cells (VSMCs) and endothelial cells (ECs). We also discuss the therapeutic potential of polyphenols in reducing the effects of the SASP and the incidence of CVD.

## 1. Introduction

Normal cell metabolism results in the generation of damaging free radicals, including reactive oxygen species (ROS) that are eliminated by antioxidant enzymes. Overtime, increased expression of ROS-generating molecules and/or reduced expression of antioxidant enzymes, like catalase, superoxide dismutases (SODs), and glutathione peroxidases (GPxs) promote the accumulation of ROS leading to damage to DNA, lipids and proteins. Accumulation of damaged molecules and downregulation of mitochondrial function, which causes oxidative stress, are associated with aging and increased incidence of age-related diseases, including cardiovascular disease (CVD), cancer, and other chronic disease states. Only recently has aging itself been viewed as a treatable condition, with an etiology related to excessive ROS levels that lead to cellular senescence, a process by which cells enter a permanent state of cell cycle arrest. Although senescent cells lost their proliferative capacity, they still are able to secrete a range of proinflammatory molecules through the senescence associated secretory phenotype (SASP).

The purpose of this review is to evaluate the most current research testing the role of foods rich in polyphenols and specific phenolic compounds in pathways upregulated during aging. This review will mainly focus on polyphenols targeting known pathways involved in senescence such as signaling pathways mediated by angiotensin II (Ang II), mitochondrial dysfunction and NADPH oxidases. An additional aim is to discuss possible mechanisms by which polyphenols exert their biological effects in senescence pathways.

## 2. Senescence

Senescence is a condition in which cells stop proliferating and become dysfunctional. Senescence can be classified in two major categories: replicative senescence and stress-induced premature senescence (SIPS) [1]. Replicative senescence is associated with shortening of telomeres, that cap the ends of chromosomes, which occurs after every cell division. This type of senescence is activated when cells exhaust their replicative capacity after several cycles of cell division and, therefore, takes time to develop. SIPS, on the other hand, develops faster and occurs in response to a stressor, like oxidative stress, radiation, cigarette smoke and oncogenes. For example, oncogene-induced senescence (OIS), caused by activation of an oncogene like Ras, prevents proliferation of tumor cells. Thus, this type of senescence is considered as a tumor suppressor mechanism [2]. Telomere attrition and stress conditions cause chromosome instability and DNA damage leading to activation of the DNA damage response (DDR) and inhibition of cell cycle progression. During activation of the DDR, the kinases ataxia telangiectasia mutated (ATM) and ataxia telangiectasia and Rad3-related (ATR) upregulate the expression of inhibitors of cell cycle progression, such as p16 [3] and p21 [4], which inhibit cyclin dependent kinases (CDKs) leading to cell cycle arrest. Both replicative senescence and SIPS are also marked by the expression of senescence-associated β-galactosidase (SA-β-gal) [5] and the tumor suppressor p53, which is an upstream regulator of p16 and p21 [6]. In some instances, stress conditions such as oxidative stress can accelerate telomere shortening and cause early onset of senescence [7]. However, telomere shortening is not always involved in SIPS. For example, treatment of human diploid fibroblast cells with hydrogen peroxide (H_2_O_2_) induces senescence by increasing the expression of p21 without shortening of telomeres [8].

Over time, senescent cells develop the SASP, secreting abnormal levels of molecules such as interleukins (ILs), matrix metalloproteinases (MMPs), monocyte chemotactic proteins (MCPs), growth factors, such as insulin-like growth factors (IGFs), and ROS [9], inducing inflammation and senescence in neighboring cells. While senescence could be seen as a tumor suppressor due to reduced proliferative capacity of the affected tissue, the SASP may stimulate proliferation of an established tumor [10]. The SASP also contributes to the spread of senescence in tissues and organs during aging, promoting low-grade inflammation and oxidative stress associated with chronic age-related diseases. In this review, we will focus on the contribution of the SASP to vascular aging and CVD.

Chronic inflammation occurs with aging as the immune system responds to an accumulation of stressors throughout life [11] and also by the secretion of proinflammatory molecules through the SASP. In senescent vascular smooth muscle cells (VSMCs), IL-1α, which is secreted through the SASP, induces senescence in neighboring cells [12]. IL-1α is central to this pathway connecting protein translation, regulated by the mammalian target of rapamycin (mTOR), and inflammation, regulated by nuclear factor-κB (NF-κB) [13]. Mechanistically, activation of mTOR during senescence stimulates translation of selected targets, including IL-1α, which then stimulates NF-κB transcriptional activity upregulating the expression of a large set of SASP components [13]. Interestingly, in this study, inhibition of mTOR with rapamycin reduced NF-κB-induced secretion of SASP components, but did not prevent senescence induced by ionizing radiation, suggesting that the mTOR/IL-1α/NF-κB/SASP pathway is downstream of the DDR. Thus, NF-κB is considered a major driver of the SASP, while inhibition of mTOR is viewed as a therapeutic target to reduce the damaging effects of the SASP. The SASP can also be induced by activation of p38 mitogen-activated protein kinase (p38 MAPK), which increases the transcription of NF-κB [14]. In this case, the p38 MAPK-dependent pathway was activated independently of the DDR since overexpression of a dominant active p38 MAPK mutant was enough to induce cell cycle arrest and to increase NF-κB expression and the SASP [14].

NF-κB plays an important role in the development of the SASP. For example, in VSMCs the NF-κB pathway leads to increased production of MMP-2 [15], a component of the SASP, which is associated with unstable atherosclerotic plaque. Our group reported that NF-κB increases the expression of the NADPH oxidase 1 (Nox1), an enzyme that produces superoxide [16]. Inhibition of NF-κB reduced Nox1 upregulation and resulted in the reduction of cellular senescence induced by zinc overload [16]. We also demonstrated that activation of NF-κB by zinc overload was mediated by mitochondrial ROS since the mitochondrial ROS scavenger MitoTEMPO (Mitochondria-targeted (2,2,6,6-tetramethylpiperidin-1yl) oxyl) reduced NF-κB activation, Nox1 expression and senescence [16]. Although, we did not measure SASP components in this study, these data suggest that the development of the SASP appears to require cellular stress, not just suppressed proliferation. In fact, senescence induced by oncogenic Ras, which causes genotoxic stress, resulted in the expression of SASP components [17], while senescence induced by overexpressing tumor suppressor p16 does not induce the SASP in human epithelial cells [18]. In favor of this view, Nelson et al. [19] showed that the bystander effect of senescent cells is caused by activation of NF-κB by mitochondrial ROS, leading to secretion of proinflammatory cytokines, including IL-6 and IL-8, that act on neighboring cells to induce senescence [19]. Altogether, these data indicate that not all senescent cells will develop the SASP, but stress induced premature senescent cells likely will.

In this review, we focus on the SASP because secretion of SASP components contribute to CVD. Chronic inflammation is a risk factor for atherosclerosis. Patients with coronary artery disease show increased expression of the proinflammatory molecules MCP-1, IL-6, IL-1β and tumor necrosis factor α (TNF-α) [20], all of which are secreted by senescent cells through the SASP. Stress from oncogene Ras induction proved to increase senescence, and proinflammatory secretions such as IL-1α, IL-1β, IL-6, IL-8, and MCP-1 in VSMCs in vitro and in vivo in rat carotid arteries [21]. These are significant findings as senescent VSMCs and these secreted molecules were also found in human plaque [21]. Furthermore, the effects of cellular stress can be seen by the increase in oxidative DNA damage and shortened telomeres in ECs and VSMCs of patients with abdominal aortic aneurism [22]. Shortened telomeres and oxidative DNA damage indicated that SIPS was the likely cause of the development of this disease. Our group also reported that oxidative stress induced by overexpression of Nox1 caused senescence of VSMCs by telomere-dependent mechanisms, as evidenced by the downregulation of telomerase, as well as telomere-independent mechanisms associated with DNA damage, as evidenced by upregulation of phosphorylated γH2AX [16]. This histone is used as a marker of DNA damage in senescent cells since it is recruited to double-stranded DNA breaks [23].

Altogether, these data suggest that reducing cellular stress will lead to a delay in the development of senescence and the SASP, thereby reducing the incidence of CVD. Knowing that NF-κB is a central regulatory node in Nox1 expression, ROS production, the development of the SASP and the bystander effect, we hypothesize that consumption of functional foods rich in polyphenols or individual phenolic compounds targeting NF-κB and/or its upstream regulators, mTOR and p38 MAPK, may be a therapeutic method to reduce the SASP and fight the onset of age-related diseases.

## 3. NADPH Oxidases in Aging

NADPH oxidase enzymes are major producers of ROS, generating species such as hydrogen peroxide and superoxide. The enzymes Nox1, Nox2, Nox3, Nox4, Nox5, dual oxidase 1 (Duox1) and Duox2 belong to the NADPH oxidase family, but differ in their location, activation, and type of ROS they produce. Each of these enzymes has been reviewed extensively by others [24,25,26]. We have also recently reviewed more specifically the contribution of Nox1 and Nox4 to vascular senescence [27].

**Figure 1:**
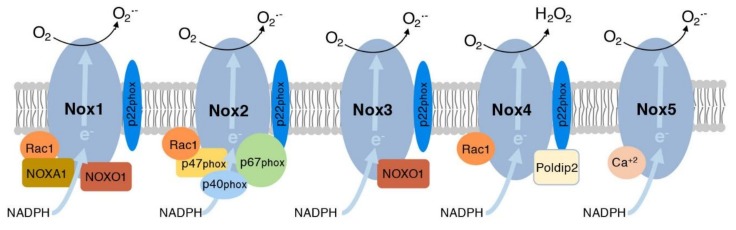
Schematic representation of the structure of the five major NADPH oxidase enzymes. These enzymes differ in the core transmembrane subunit. Similarities among these enzymes include the presence of the p22phox membrane subunit in Nox1–Nox4 and interaction with the small GTPase Rac 1 that regulates the activity of Nox1, Nox2, and Nox4. Nox1 contains NOXA1 and NOXO1, while Nox2 contains p47phox, p40phox and p67phox. Nox3 is constitutively active and interacts with NOXO1. In contrast, Nox4 interacts with Rac1 and is regulated by Poldip2, while Nox5 is regulated by calcium. All these enzymes take an electron from NADPH to generate superoxide, except for Nox4 that generates H_2_O_2_.

Nox1, Nox2, Nox4, and Nox5 (Figure 1) are the only Nox enzymes found in blood vessels. They can be found in endothelial cells (ECs) in the intima, VSMCs in the media or in the adventitia. Nox1 is commonly found in the plasma membrane and endosomes of vascular ECs, VSMCs, and adventitial fibroblasts [24]. It is activated by Ang II [28,29] and platelet derived growth factor (PDGF) [30], and requires the stabilizing subunits Nox activating protein 1 (NOXA1), Nox organizer 1 (NOXO1), and p22phox, as well as the small GTPase Rac1 (Ras-related C3 botulinum toxin substrate 1), to activate superoxide production [31]. Similarly, Nox2 requires Rac1, p22phox, p47phox, p40phox, and p67phox to be active [31]. This enzyme produces superoxide upon activation by proinflammatory cytokines, like IL-17 and interferon γ (IFNγ) [32,33]. Similar to Nox1, Nox2 is found in ECs, adventitial fibroblasts, and VSMCs [24]. Nox5 also secretes superoxide and it is regulated by calcium; however, its mechanism of activation is not well known because it is only found in human vasculature and cannot be studied in rodent models [24]. Nox4 is unique in that it is the only vascular Nox enzyme that does not produce superoxide. It produces H_2_O_2_ and it is constitutively active. Different from superoxide, H_2_O_2_ can cross cellular membranes of intracellular organelles and the plasma membrane affecting neighboring cells as well. Nox4 requires the subunits Rac1, p22phox, and polymerase delta-interacting protein 2 (Poldip2) for activation [31]. It is usually found in the endoplasmic reticulum, focal adhesion, and nucleus of VSMCs [34]. Thus, activation of each of these enzymes depends on the expression and recruitment of regulatory subunits to cellular membranes. In contrast, regulation of Nox3 activity by cytosolic regulators is less clear. In overexpression experiments, Nox3 showed constitutive generation of superoxide that required p22phox, NOXA1 and NOXO1, but not Rac1 [35]. Nox3 is highly expressed in the inner ear and lack of this enzyme in Nox3^−/−^ mice showed defects in balance [36]. This balance defect was not recapitulated in NOXA1^−/−^ mice [37], suggesting that NOXA1 is not required for Nox3 function in vivo. Additionally, Nox3 was shown to be upregulated in conditions of stress induced by TNF-α in HepG2 cells, suggesting a role for Nox3 in insulin resistance in the liver [38].

This review focuses mainly on the inhibitory effect of polyphenols in both the expression and activity of Nox1, Nox2 and Nox4. We focused on these enzymes because they have been involved in the induction of senescence in ECs, as well as in VSMCs [27]. When looking at the function of Nox enzymes, it is important to consider that these enzymes may co-regulate each other. For example, H_2_O_2_ was shown to activate NADPH-dependent superoxide production in VSMCs [39]. Thus, Nox4 may activate Nox1 and Nox2. Similarly, the ability of Nox4 to produce H_2_O_2_ depends on critical cysteine residues in an extracellular loop [40]. Thus, oxidation of these residues by Nox1- or Nox2-dependent superoxide production could change the function of Nox4 from a H_2_O_2_ to a superoxide generating enzyme.

## 4. Mitochondrial Function in Aging

A major source of ROS production comes from the mitochondria as a byproduct of the electron transport chain. The electron transport chain is the body’s main producer of energy through generation of ATP by the enzyme ATP synthase, which is powered by a proton gradient. Electrons are transferred through complexes of the electron transport chain to create this gradient, but during this process some electrons react with oxygen molecules producing superoxide [41]. As mentioned previously, Nox enzymes can regulate each other, but these enzymes can also regulate mitochondrial oxidative stress. In fact, mitochondrial ROS production was upregulated by Nox4, which targeted complex I of the electron transport chain [42]. Similarly, mitochondrial ROS can upregulate Nox function. Our group showed that mitochondrial ROS contributes to senescence by activating NF-κB [16]. NF-κB is a transcription factor that regulates the expression of both Nox1 and Nox4 [43]. Inhibition of mitochondrial ROS with MitoTEMPO reduced NF-κB activity, Nox1 expression and ROS production. Therefore, mitochondrial ROS leads to increased Nox1 levels, ROS production and senescence through activation of NF-κB.

Mitochondrial dysfunction leading to mitochondrial ROS production acts as an inducer of senescence, but depending on the stimulus, mitochondrial dysfunction is also a consequence of senescence. Telomere attrition upregulates p53 expression, which leads to a decrease in the expression of peroxisome proliferator-activated receptor γ coactivator-1α (PGC-1α) [44]. PGC-1α is a transcriptional coactivator responsible for the regulation of mitochondrial biogenesis, as well as expression of antioxidant enzymes [45]. Antioxidant enzymes react with ROS to convert them into other molecules, for example, SOD1 and SOD2 convert superoxide into H_2_O_2_, which is then converted to water by catalase and GPxs. Reduced PGC-1α expression resulted in a decrease in the antioxidant enzymes SOD2, thioredoxin 2 (TRX2), thioredoxin reductase 2 (TRXR2), peroxirredoxin 3 (Prx3) and Prx5 in the mitochondria [46], which further contributed to the increase of mitochondrial ROS. Thus, telomere dysfunction causes impaired mitochondrial function by a PGC-1α-dependent mechanism. On the other hand, reduced PGC-1α expression or function leading to mitochondrial dysfunction is sufficient to induce senescence. Our group demonstrated that reduced PGC-1α activity mediated by Akt-dependent phosphorylation and acetylation of PGC-1α, in response to Ang II [47], increased ROS levels that caused senescence in VSMCs [48]. Furthermore, PGC-1α^−/−^ VSMCs showed increased DNA damage and telomere attrition [45]. Importantly, PGC-1α also stimulated the expression of the longevity gene Sirt1 (silent information regulator 2 homologue 1). Downregulation of either PGC-1α or Sirt1 induced senescence, while their overexpression reduced Ang II-induced senescence [48]. However, it is not known whether PGC-1α may also regulate the expression of Nox enzymes, like Nox1, which is upregulated by mitochondrial ROS [16]. Altogether, polyphenols may regulate Nox enzymes indirectly by targeting PGC-1α and/or Ang II signaling.

## 5. Polyphenols in Human Health

There are many classes of polyphenols but all have the common feature of having at least one aromatic ring with a hydroxyl group attached to it. In plants, polyphenols promote plant survival by acting as pollinators, protecting against ultraviolent radiation, and scavenging ROS [49]. Some of these protective benefits seem to transfer to human health as well, promoting longevity by decreasing the incidence of chronic diseases.

Many dietary strategies, summarized in Table 1 and Table 2, have been reported to slow the progression of CVD by improving lipid profile, reducing the generation of ROS and by enhancing the body’s own antioxidant capacity. Lai et al. [50] reported that total fruit intake (based on food frequency questionnaire (FFQ)), the richest sources of phenolic compounds, correlated with improved cardiovascular health with an estimated 6–7% reduction in deaths from CVDs for every 80 g portion. This study also concluded that total fruit intake rather than intake of a specific type of fruit is protective against CVD. In terms of specific types of fruits, commonly researched varieties include berries, such as blackberries, raspberries, black raspberries and blueberries [29,51], grapes [52,53], citrus fruit [50], pomegranates [54,55], strawberries [56] and apples [57,58,59]. These fruits are rich in polyphenols like flavanols, flavonols, anthocyanins, procyanidins, sterols, carotenoids, and hydroycinnamic acids [50].

Since fruits are also rich in other components, such as vitamins and fiber, it is difficult to conclude that polyphenols were the active ingredients in these studies. It is possible that in the context of the whole fruit, fiber could be important for the proper function of bacteria in the gut, which are known to metabolize polyphenols into active compounds. Ravn-Haren et al. [58] compared the effect of whole apple (WA), apple pomace (AP) and apple juice (AJ) and found that WA and AP, but not clear AJ reduced total cholesterol (TC) and low-density lipoprotein (LDL) cholesterol in healthy volunteers. Although authors concluded that fiber in WA and AP mediated the effect of apple in cholesterol levels, the effect of fiber in the gut microbiota cannot be ruled out.

To address more specifically the role of polyphenols, many studies have used purified polyphenol extracts in animal models of human diseases. For example, Yang et al. [62] supplemented polyphenol extracts of the sea buckthorn berry to rats fed a high fat diet (HFD) and found that the extract reduced TNFα and IL-6 levels, while increasing the activity of antioxidant enzymes, compared to HFD alone [62]. These findings indicate that polyphenols can reduce inflammation and increase the antioxidant capacity, both of which may provide protection against atherosclerosis. These protective effects were seen with many other fruits as well. A diet supplemented with 1% blueberry reduced atherosclerotic lesion area in the aortas of 4-month-old ApoE^−/−^ mice, compared with placebo. This effect was associated with increased antioxidant activity of SOD1, SOD2 and GRx and reduced lipid peroxidation [66]. Blueberry consumption also decreased peripheral artery dysfunction in both smoking and non-smoking male subjects 2 h after consuming 300 g of blueberries [80]. Strawberries have shown to have beneficial effects in patients with Type II diabetes (T2D) and metabolic syndrome. In female patients with metabolic syndrome, 4 cups of freeze-dried strawberries (FDS) consumed daily for 8 weeks, decreased LDL-cholesterol and the levels of vascular cell adhesion molecule 1 (VCAM-1) [69]. Similarly, in another study, consumption of 2 cups of FDS daily for 6 weeks lead to a decrease in the levels of hemoglobin A1c (HbA1c), as well as C-reactive protein (CRP), lipid peroxidation, and an increase in total antioxidant status in female patients with T2D [68]. These data suggest that in females, FDS can reduce the risk factors of atherosclerosis.

Fruits are not the only food rich in polyphenols that have shown beneficial effects. For example, 200 g of purple majesty potatoes, containing 288 mg of anthocyanins, decreased pulse wave velocity, a measure of arterial stiffness, in both healthy males and females consuming 200 g of potatoes a day for 14 days [73]. Since arterial stiffness is an independent indicator of CVD, purple majesty potatoes consumption would be beneficial against these diseases. Extra virgin olive oil (EVOO) is another food rich in polyphenols (Table 2). EVOO reduced Nox2 activity and H_2_O_2_ levels to a similar degree as catalase in platelets isolated from blood of healthy human subjects [78]. It is known that polyphenols are the active component of EVOO because when enriched with its own or other polyphenols, EVOO increased high density lipoprotein (HDL) levels, compared with non-enriched EVOO [79]. Thus, all fruits, vegetables and EVOO discussed so far seem to be protective against CVD by reducing LDL, cholesterol, triglycerides, inflammatory molecules and arterial stiffness; and by increasing the antioxidant capacity and HDL levels. Although polyphenols are the main candidates for these protective effects, other nutrients like vitamins, minerals and fiber may also contribute to the effects of these foods, as previously discussed. Polyphenols can be found in a variety of foods and other examples of their beneficial effects can be found in Table 1 for fruits and Table 2 for vegetables and olive oil.

## 6. Regulation of NADPH Oxidases by Polyphenols

### 6.1. Structural Elements in Polyphenols Involved in NADPH Oxidase Function

**Figure 2:**
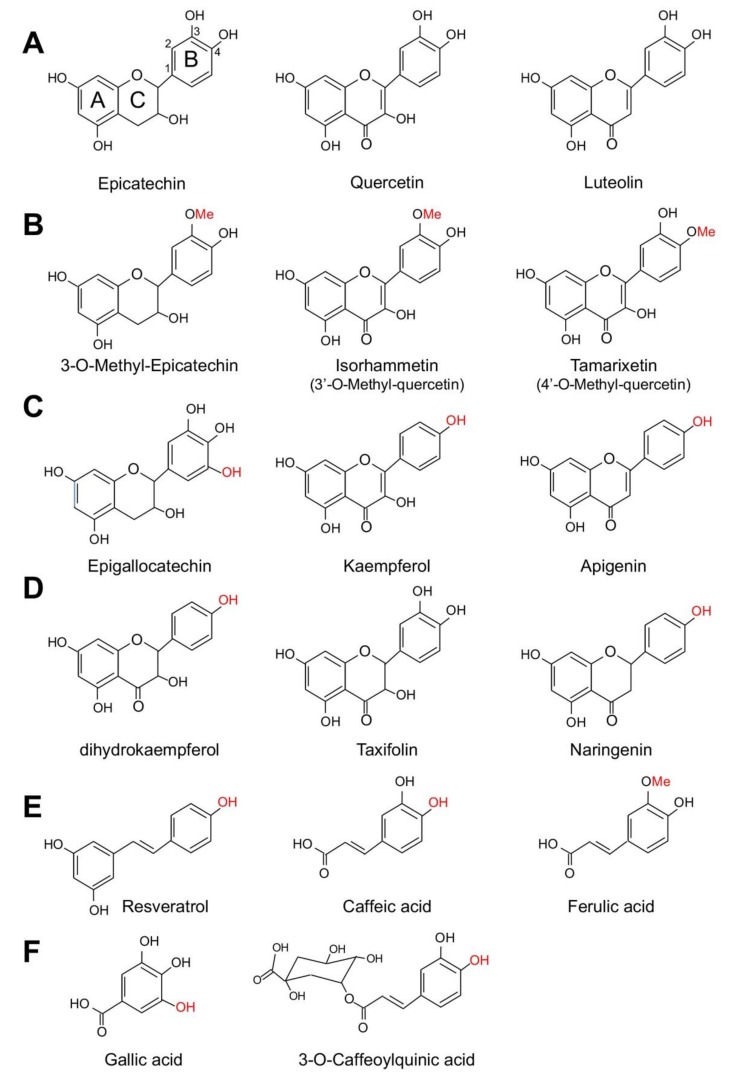
Structure of major polyphenols involved in the regulation of NADPH oxidase activity and expression. (**A**) Shows the structure of unmodified cathechin; (**B**–**E**) show hydroxylated and O-methylated compounds shown to affect NADPH oxidase activity in several cells types and treatment conditions; (**F**) shows the structure of two phenolic compounds found in blackberry polyphenol extract that we hypothesize could modulate NADPH oxidase activity and Nox1 expression.

Polyphenols can be classified according to their chemical structure [81] (Figure 2) into three groups: (1) phenolic acids, which are non-flavonoid compounds, including gallic acid, caffeic acid and ferulic acid (Figure 2E,F). This family contains one C6 carbon phenolic ring. (2) Flavonoids, which contain two C6 phenolic rings (ring A and ring B, Figure 2A) with ring A binding to a chromane ring. Members of this family can be subdivided into different groups according to modifications in ring C, such as addition of double bonds in isoflavones; addition of hydroxyl groups to ring B, like in the case of the flavonols, quercetin and kaempferol; lack of double bonds and a carbonyl group in ring C, like in the case of catechins and epicatechins; and hydroxylation and methoxylation of ring B, for example in anthocyanidins, like malvidin and cyanidin. (3) Non-flavonoids, which include resveratrol, found in grapes, curcumin, found in turmeric, and ellagic acid, found in berries.

**Figure 3:**
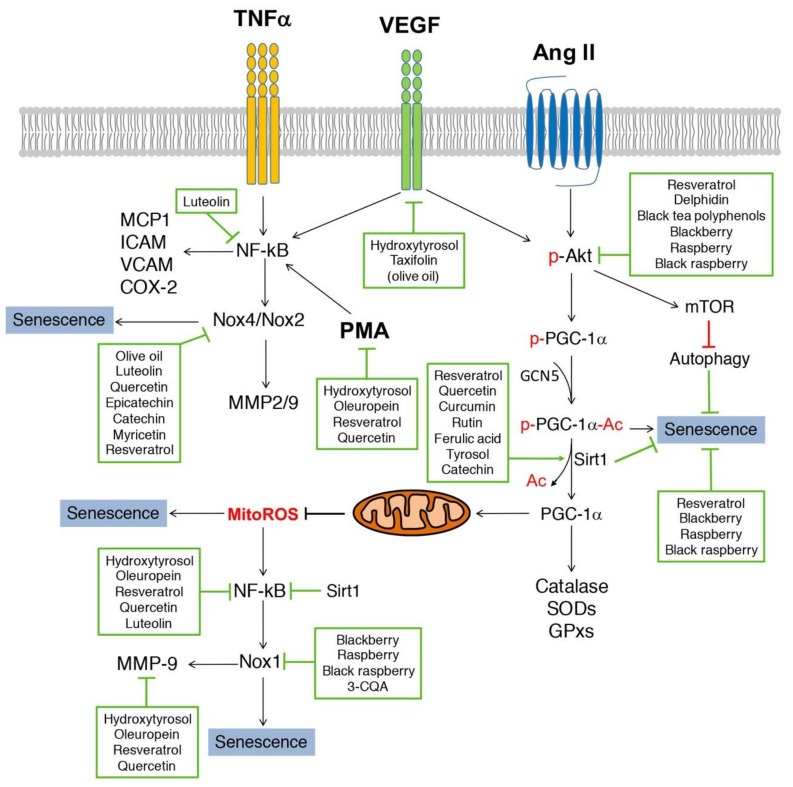
Proposed model by which polyphenols may modulate oxidative stress-induced pathways including Ang II, VEGF and TNFα. These inflammatory pathways activate common signaling pathways associated with Akt/mTOR/PGC-1α and NF-κB/Nox(s)/MMP pathways. Polyphenols from fruits, vegetables and olive oil inhibit these signaling pathways at different levels. Overall, polyphenols found in the Mediterranean diet reduce inflammation and oxidative stress pathways associated with senescence. Mitochondrial ROS (MitoROS), which is upregulated during aging and by activation of Ang II signaling pathway is also reduced by polyphenols.

**Figure 4:**
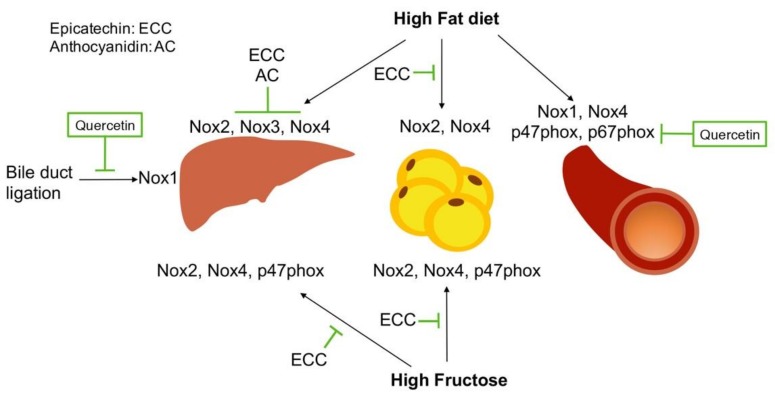
Inhibition of NADPH oxidases in different organs in vivo mediate the protective effects of polyphenols. In animal models in vivo, high fat diet and high fructose diet increase the expression of NADPH oxidases in the liver, adipose tissue and aorta of mice. The polyphenols, epicatechin, anthocyanidin and quercetin have shown protective effects in these tissues by downregulating Nox1, Nox2, Nox3 and Nox4.

A study by Steffen et al. [82] tested the role of several polyphenols in oxidative stress in ECs. This group established a set of structural requirements for scavenging of ROS and inhibition of NADPH oxidase function as follows: (1) superoxide scavenging activity is mediated by flavonoids lacking additional substitutions in ring B. Polyphenols in this category included catechin, epicatechin, quercetin, luteolin and fisetin (Figure 2A); (2) inhibition of NADPH oxidase activity requires additional substitutions in ring B. For example, O-methylation in ring B (3-O-Methyl-epicatechin, isorhammentin and tamarixerin, Figure 2B), the presence of a 4′-OH group in ring B (kaempferol and apigenin, Figure 2C) and the addition of an extra OH group in ring B as in the case of epigallocatechin (Figure 2C); (3) Hydrogenation of the C2-C3 double bond in ring C, like in the case of dihydrokaempferol, taxifolin and niringenin (Figure 2D); (4) also, it seems that the presence of a OH or methyl group in an aromatic ring also provided inhibitory effects in NADPH oxidase activity as seen for resveratrol, caffeic acid and ferulic acid (Figure 2E) and for gallic acid and 3-O-caffeoylquinic acid (3-CQA) (Figure 2F). This in vitro evidence is supported by in vivo studies. For example gallic acid reduced Nox2 expression in the heart of mice infused with Ang II [83]. Gallic acid also reduced the expression of p47phox, NF-κB, TNFα, MMP-2 and MMP-9 in rats infused with advanced glycation end products [84], thus reducing cardiac fibrosis. The putative role of 3-CQA in Nox function will be discussed in a later section in the review. Based on these structural elements, we will discuss in the following sections the role of some of these polyphenols in regulating NADPH oxidase activity and function in cells in culture, including ECs and VSMCs, summarized in Figure 3. Ang II, VEGF and TNFα stimulate signaling pathways that promote senescence, for example VEGF and TNFα increase NF-κB activity leading to upregulation of Nox2 and Nox4 function. These Nox enzymes then upregulate the expression of the SASP components MMP-2 and MMP-9. Ang II on the other hand, causes senescence by multiple mechanisms, including activation of the Akt/mTOR pathway leading to reduced autophagy; inhibition of PGC-1α function leading to reduced antioxidant capacity and increased mitochondrial ROS; and upregulation of NF-κB and Nox1 expression. The fact that NF-κB is a mediator of these pathways suggest that the SASP is a major mediator of senescence in these conditions. The effect of various phenolic compounds in the pathways shown in Figure 3 will be discussed in the following sections.

### 6.2. Mediterranean Diet: Extra Virgin Olive Oil and Grapes

Olive oil is an important component of the Mediterranean diet, which is associated with reduced mortality from CVD, in part by reducing oxidative stress and inflammation [85]. Several studies have demonstrated the protective effect of olive oil extracts and their individual phenolic compounds in ECs. Calabriso et al. [86] reported that 1–10 μg/mL of olive oil polyphenol extract reduced the angiogenic response of ECs to vascular endothelial growth factor (VEGF). This effect was associated with reduced NADPH oxidase activity, ROS levels, and Nox2, Nox4, MMP-2 and MMP-9 expression. This report, however did not demonstrate whether Nox2 and/or Nox4 were required for the modulation of these metalloproteinases. On the other hand, the olive oil polyphenol luteolin reduced Nox4 and p22phox expression in response to TNFα in ECs [87]. Luteolin was also shown to inhibit monocyte adhesion to ECs by suppressing NF-κB and the expression of the inflammatory marker MCP1, the adhesion molecules intercellular adhesion molecule-1 (ICAM-1) and VCAM-1 [88]. The fact that these molecules are also part of the SASP, suggest that olive oil polyphenols may reduce the SASP to alleviate senescence. Lamy et al. [89] also tested the role of the polyphenols tyrosol, hydroxytyrosol, taxifolin and oleuropein, as well as the omega-9 fatty acid oleic, found in olive oil, in the angiogenic response of ECs to VEGF. Interestingly, hydroxytyrosol, taxifolin and oleic acid inhibited the phosphorylation of the VEGF receptor 2 preventing signaling through this receptor. Additionally, hydroxytyrosol inhibited protein kinase C α (PKCα) and PKCβ to reduce MMP-9 and COX-2 expression in human monocytes in response to phorbol myristate acetate (PMA) [90]. In vivo studies also showed the protective role of olive oil in reducing oxidative stress and Nox2 expression in platelets [91]. Olive oil polyphenol extract also protected ECs from high glucose and free fatty acids by increasing nitric oxide (NO) and reducing endothelin-1 levels [92].

Scoditti et al. [93] compared the effects of the olive oil polyphenols hydroxytyrosol and oleuropein with the polyphenols resveratrol and quercetin found in grapes. Grapes and red wine, rich in resveratrol, are also important components of the Mediterranean diet. 10 μM olive oil polyphenols and 1 μM grape polyphenols reduced the angiogenic response of ECs to PMA. MMP-9, which is upregulated by PMA, and it is also regulated by Nox1 [94], was reduced by all four phenolic compounds. Inhibition of MMP and NF-κB, a transcriptional regulator of COX-2 and MMP-9, prevented PMA-induced migration. The fact that COX-2 expression is modulated by NADPH oxidases [95], and that MMP-9 is regulated by Nox1 suggest that these phenolic compounds inhibit a NADPH oxidase to reduce endothelial inflammation, oxidative stress and migration. In fact, red grape juice [96], as well as resveratrol [97] inhibit the expression of NADPH oxidases. Red grape juice extract and its polyphenols quercetin, (−)-epicatechin, (+)-catechin and myricetin reduced NADPH oxidase activity in neutrophils, which was associated with reduced expression of p22phox, p47phox and gp91phox/Nox2. Among all polyphenols tested, quercetin was the most effective. Similar effects were observed with red wine polyphenol extracts [96]. Interestingly, this group showed that ascorbic acid was ineffective in reducing NADPH oxidase activity, suggesting that the antioxidant capacity of polyphenols was not involved in the regulation of NADPH oxidase activity or expression. In contrast in ECs, red grape juice extract, quercetin, and red wine polyphenol extract only reduced the expression of p47phox, but not p22phox, p67phox or gp91phox/Nox2. Overall, this study showed that grape juice polyphenols reduced superoxide levels by downregulating NADPH oxidase activity and/or expression.

In the case of resveratrol, Spanier et al. [97] showed that 10 to 100 μM resveratrol for 24 h reduced Nox4, while increasing SOD1 and GPx1 mRNA levels in human umbilical ECs (HUVEC) and the endothelial cell line EA.hy926, which was associated with reduced ROS levels. In contrast to this study, Schilder et al. [98] showed that chronic exposure of HUVEC with 10 μM resveratrol for several passages reduced the proliferative capacity of ECs by inducing senescence. In this study, treatment with 10 μM resveratrol for 24 h increased ROS levels, which was prevented by downregulation of either Nox1 or Nox4. The contradicting effect of resveratrol in these studies is not known.

Recent research supports a protective role of resveratrol in vascular senescence in vitro and in vivo. In bovine aorta ECs (BAECs) 1 μM resveratrol reduced glucose-induced senescence by downregulating p47phox expression and SA-β-gal activity and by increasing Sirt1 expression [99]. Importantly, inhibition of Sirt1 activity prevented the effect of resveratrol in p47phox, suggesting that NADPH oxidases are regulated by a Sirt1-dependent mechanism. Additionally, resveratrol was shown to upregulate autophagy by inhibiting the Akt/mTOR pathway in different cell types including non-small-cell lung cancer cells [100], nucleus pulposus cells [101] and adenocarcinoma-derived MCF7 cells [102]. In VSMCs, resveratrol (1) prevented Akt activation in response to Ang II and epidermal growth factor (EGF) [103]; (2) stimulated differentiation by activating Sirt1 and AMPK [104]; (3) reduced Akt-induced hypertrophy [105]; and (4) reduced Ang II-induced senescence [106]. Similarly, black tea polyphenols inhibited Akt activation induced by IGF in prostate epithelial cells [107], and delphidin, a red wine anthocyanin, inhibited Akt activation in ECs in response to VEGF [108]. The effect of delphidin was associated with upregulation in the expression of nuclear respiratory factor 1 (Nrf1), estrogen-related receptor-α (ERRα) and the mitochondrial transcription factor TFAM. Although, not all of these studies evaluated the role of polyphenols in autophagy, the fact that Akt activity was inhibited suggests that autophagy should be upregulated. Autophagy is a protective pathway in which dysfunctional proteins and organelles are degraded. Autophagy is downregulated during aging and inhibition of this pathway causes senescence and is associated with CVD [109].

### 6.3. Polyphenols in Mitochondrial Function

The protective role of resveratrol in mitochondrial function is well established and it is mainly mediated by the activation of Sirt1 and the subsequent deacetylation (activation) of PGC-1α leading to improved mitochondrial biogenesis and function and increased expression of antioxidant enzymes. A recent review on polyphenols in mitochondrial function summarizes studies looking at the effects of resveratrol, curcumin, quercetin and epigallocatechin gallate in the Sirt1/PGC-1α axis [110]. Resveratrol was shown to extend life span in mice on high-calorie diets, which was associated with improved insulin sensitivity, and increased expression of AMPK and PGC-1α [111]. Similarly, resveratrol protected mice from diet-induced obesity and insulin resistance and improved aerobic capacity by increasing oxygen consumption in the muscle [112]. In older subjects, resveratrol was also shown to improve vascular function and mitochondrial mass [113]. Other polyphenols have also been shown to increase the Sirt1 pathway, including rutin and quercetin glycoside, which upregulated Sirt1 in the kidney alleviating kidney injury [114]. Quercetin also upregulated the AMPK/Sirt1 pathway in a model of osteoarthritis in rats [115]. Finally, in a study using HeLa cells, Sirt1 was upregulated by quercetin, ferulic acid, berberine, curcumin, tyrosol and catechin [116].

### 6.4. Catechins in NADPH Oxidases

(−)-Epicatechin, a polyphenol found in fruits, vegetables and tea, has protective effects in many conditions including insulin resistance [117] and atherosclerosis [118,119]. The protective effect of tea polyphenols in cardiovascular health was recently reviewed by others [120,121]. Detailed information on the role of catechins in oxidative stress defense mechanism was also reviewed recently [122]. The effect of these polyphenols in the liver, adipose tissue and blood vessels is summarized in Figure 4. Stress conditions, such as HFD and high fructose diet increase the expression of Nox1, Nox2, Nox3 and Nox4 in the liver, adipose tissue and blood vessel in mice. Polyphenols, as described below, show protective effects in these tissues by reducing the expression of these enzymes and their regulatory subunits.

In the liver, Cremonini et al. [117] recently reported that epicatechin reduced Nox3 and Nox4 expression, two major NADPH oxidases found in the liver, in mice fed HFD. This group confirmed these observations in HepG2 cells in culture treated with palmitate. Epicatechin reduced palmitate-induced Nox3 and Nox4 upregulation, oxidative stress and activation of the C-Jun N-terminal kinase (JNK). This group also reported that a high fructose diet increased Nox2 and Nox4 in the liver and adipose tissue [123], while a HFD increased Nox2 and Nox4 in adipose tissue in mice [124]. All these effects were reduced by epicatechin supplementation. Similarly, an anthocyanidin blend rich in cyanidine and delphinidin attenuated the upregulation of Nox3 and Nox4, but not Nox2 induced by HFD in the liver [125]. Furthermore, in the small intestine HFD increased the expression of Nox1 and Nox4 and cell signaling through NF-κB and ERK (extracellular signal-regulated kinase), effects that were also reduced by epicatechin supplementation [126]. Although these studies also showed increased inflammation and NF-κB activation, it is unknown whether NF-κB, a transcriptional regulator of NADPH oxidases, mediated the upregulation of these enzymes [43] and whether epicatechin acts directly on NF-κB to reduce the effects of the high fat and fructose diet.

In the vascular system, epicatechin was shown to improve NO availability in arteries in vivo and to protect HUVECs from oxidative stress induced by Ang II in vitro [82]. Interestingly, this study showed that epicatechin metabolites, produced by ECs, have different effects on NADPH oxidase function. While epicatechin acted as a superoxide scavenger, metabolite like 3′- and 4′-O-methyl epicatechin reduced NADPH oxidase activity and superoxide levels. The observation that epicatechin metabolites could also be produced by ECs suggests that polyphenol metabolism in the endothelium would be of great importance in vivo. Potentially, these metabolites could also act in VSMCs in the media and in macrophages that infiltrate blood vessels during disease conditions. Additional polyphenol compounds tested in this study that inhibited NADPH oxidase activity were epigallocatechin, the quercetin metabolite quercetin glucuronide, ferulic acid, caffeic acid, vanilic acid and resveratrol. However, the specific Nox enzymes targeted by these compounds were not identified.

Quercetin has also been involved in NADPH oxidase regulation in vivo. For example, in a rat model of bile duct ligation (Figure 4), quercetin was shown to alleviate liver injury, which was associated with downregulation of Nox1 and Rac1 [127]. In ApoE^−/−^ mice, quercetin reduced atherosclerosis (Figure 4), which was associated with reduced translocation of p47phox to the plasma membrane, reduced expression of p47phox, p67phox and Nox4 [128]. Thus, as summarized in Figure 4, polyphenols have a broad effect in different tissues and organs. Metabolism of polyphenols in these tissues are likely different and may account for the differential effects of these nutrient in specific Nox enzymes.

In VSMCs, our group showed that polyphenol extracts from blackberry, raspberry and black raspberry reduced NADPH oxidase activity induced by Ang II [29]. The strongest effect was observed for blackberry, which also reduced Nox1 expression. The extract of this berry is rich in epicatechin and epigallocatechin, which could mediate the downregulation of NADPH oxidase activity. Similarly, the raspberry extract is also rich in these two polyphenols, but this extract only reduced NADPH oxidase activity, not Nox1 expression. In contrast, the black raspberry extract contains ferulic acid and high levels of epigallocatechin that could mediate the effects of this berry. Although the expression of these polyphenols may explain the effect of these berries in NADPH oxidase activity, it is unclear which compound mediates the downregulation of Nox1 induced by the blackberry extract. By contrast with raspberry and black raspberry extracts, the blackberry extract contains higher levels of gallic acid and 3-CQA (see structures in Figure 1F). The similarity in structure between the aromatic ring in gallic acid and epigallocatechin suggests that gallic acid may be involved in regulating Nox1 activity, as mentioned previously. In contrast, the presence of a quinic acid in 3-CQA could provide an additional structural element needed to regulate Nox1 expression. It remains to be elucidated if this is in fact the case. If this is the case, 4-O-caffeoylquinic acid and 5-O-caffeoylquinic acid found at lower levels in the blackberry extract should also be involved in the downregulation of Nox1. The effect of these polyphenols in the expression of other NADPH oxidases such as Nox2 and Nox4 remains to be tested. It is also unknown whether VSMCs are able to metabolize polyphenols similarly to ECs in culture.

In terms of Ang II signaling, our group demonstrated that inhibition of either, Akt, p38 MAPK or ERK1/2 reduced Ang II-induced senescence [29], suggesting that polyphenols inhibiting Akt phosphorylation, like delphidin and black tea polyphenols (Figure 3) should also reduce senescence.

Finally, as summarized in Figure 3, several polyphenols inhibited the expression and/or activity of signaling pathways involved in senescence, however the exact mechanism of this inhibition requires further research. For instance, polyphenols may inhibit upstream regulators of these molecules. For example, resveratrol activates Sirt1 indirectly by inhibiting a phosphodiesterase leading to increased levels of cAMP, activation of AMPK and increased NAD+ levels that activate Sirt1 [129]. It is also unknown whether these polyphenols may inhibit the expression of Nox enzymes by epigenetic modifications, such as methylation of the DNA or posttranslational modifications to histone tails. Sirt1 for example, a histone deacetylase (HDAC), inhibits NF-κB transcription by deacetylating histone tails in the NF-κB promoter and NF-κB itself [130]. NF-κB activity is upregulated by acetylation mediated by acetyl transferases, like p300, which is also inhibited through deacetylation by Sirt1 [131]. Other polyphenols regulating NADPH oxidase function like caffeic acid, gallic acid, quercetin and myricetin were also shown to regulate HDAC activity [132]. Thus, the role of polyphenols in epigenetic regulation of Nox enzymes is an exciting area of research that needs further exploration. In addition to all these possible effects, polyphenols may also act as senolytic drugs, which specifically eliminate senescent cells, as suggested in a recent review by Gurau et al. [133]. However, the studies discussed in this review suggest that polyphenols act as anti-SASP drugs by targeting NF-κB, thus, inhibiting the bystander effect of the SASP.

## 7. Conclusions

Altogether, this review of literature provides a detailed overview of structural elements in polyphenols that could be involved in the regulation of NADPH oxidase activity and expression. Polyphenols rich in fruits, vegetables and olive oil regulate major inflammatory and ROS-dependent signaling pathways associated with senescence, which support the protective role of these foods in reducing the burden of CVD. Interestingly, many of these compounds also target NF-κB, a major driver of the SASP, suggesting their possible role as anti-aging drugs. Understanding of the molecular mechanisms involved in these protective effects may lead to better therapeutic strategies. For instance, a combination of polyphenols targeting the NF-κB/Nox/SASP pathway, the Akt/mTOR autophagy pathway, and the PGC-1α/mitochondrial pathway may provide a better protection against stress conditions that cause CVD during aging. The use of several polyphenols targeting these diverse pathways in combination with prescribed drugs could potentially reduce the dose of those medications, reduce side effects, and improve health, in particular in older populations affected by CVD.

## Figures and Tables

**Table 1:** Effect of various fruits in reducing inflammation and oxidative stress.

Author, Year	Treatment	Model	Result	Conclusion
Lai et al. 2015 [50]	Report of total fruit intake using food frequency questionnaire (FFQ) over an average of 16.7 years	30,458 women (35–69 years old)	Total fruit intake was associated with reduced risk for cardiovascular disease (CVD) mortality	Fruit intake shows health improvements in women for CVD and stroke
Zunino et al. 2014 [53]	46 g of grape powder or placebo twice daily for 3 weeks.	24 obese (8 men, 16 women; 20–60 years old)	Significant decrease in low-density lipoprotein (LDL) cholesterol and increase in immune cell function	Grapes may improve cholesterol levels and immune response in obese individuals
Ravn-Haren et al. 2013 [58]	550 g/day WA, 22 g/day AP or 500 mL/day of either clear or cloudy AJ	5 × 4 weeks cross-over study in 23 healthy volunteers	Significant decrease in total cholesterol (TC) and LDL cholesterol in WA and AP but not AJ	The fiber content in WA and AP mediates the effect of apples in cholesterol levels
Huebbe et al. 2012 [60]	Blackcurrant juice compared to red grape, raspberry, and cherry juice In vitro 0.2 mg/mL for 4 h In vivo 250 g fruit beverage with high energy meal	In vitro murine macrophage RAW264.7 In vivo human subjects with atherosclerosis prone phenotype 11 men (mean age 37.4)	In vitro, decreased TNFα, IL-1β and iNOS mRNA levels In vivo, increased radical-scavenging capacity and decreased triglycerides	Blackcurrant juice may reduce atherosclerosis by decreasing triglycerides and inflammation related to NF-κB
Karlsen et al. 2010 [61]	In vivo, 4 weeks treatment with 330 mL/day bilberry juice (BBJ) In vitro BBJ purified polyphenols	In vivo, 62 human subjects with at least one CVD risk factor. In vitro, human monocytic cell line stimulated with LPS	In vivo reduction in CRP, IL-6, IL-15 In vitro epicatechin, and resveratrol strongly inhibited NF-κB activation.	BBJ decreased NF-κB activity in individuals at high risk of CVD. In vitro polyphenols inhibited NF-κB
Yang et al. 2016 [62]	Polyphenol extract of sea buckthorn berry administered orally. 7, 14, or 28 mg/kg for 5 weeks	4-week old male Sprague Dawley rats with hyperlipidemia from high-fat diet (HFD)	Reduced serum lipids, TNFα, IL-6, eNOS, and intercellular adhesion molecule-1 (ICAM) and increased superoxide dismutases (SOD) and glutathione peroxidases (GPx) activity	Sea buckthorn berry polyphenols reduce inflammation and increase antioxidant capacity
Rosenblat et al. 2015 [55]	Consumption of 0.5 μmol gallic acid/day from pomegranate juice, Hallawi or date seed extract, or a combination for 3 weeks	6-week old male ApoE^−/−^ mice	Combination treatment decreased total triglycerides, and oxidative stress, and lipid peroxidation in macrophages in aortas	The most effective reduction of lipids and oxidative stress was found by using a combination of polyphenols from various sources
Stirpe et al. 2017 [63]	Annurca apple extract (1 and 10 mg/mL) for 3 days	S. cerevisiae strain MCY4/Kllsm4Δ1	Significant decrease in reactive oxygen species (ROS) levels and DNA damage and increased viability	Apple extract increases lifespan by reducing oxidative stress
Bravo et al. 2016 [64]	Cells treated with <10 μg/mL of *Passiflora tarminiana* extract (620 mg po-lyphenol/g for 24 h	Human diploid fibroblasts treated with UV radiation	Reduced collagen degradation, matrix metalloproteinase (MMP-1) expression and ROS levels	*P. tarminiana* reduces UVB photoaging in human skin fibroblasts
Stroher et al. 2015 [65]	Supplementation of hyperlipidemic diet with 25 or 50 mg/kg blueberry extract for 14 days	36 one-month old male Wistar rats	Reduced aortic lesions, oxidative damage and increased antioxidant capacity	Blueberry extract improved lipid profile, antioxidant defense, and reduced oxidative stress
Wu et al. 2010 [66]	AIN-93G high-fat diet supplemented with 1% freeze dried blueberry for 20 weeks	4-month old female ApoE^−/−^ mice	Decreased lipid peroxidation and atherosclerotic lesions and increased antioxidant capacity	Blueberry reduced oxidative stress and protected against atherosclerosis
Alarcon et al. 2015 [67]	In vitro platelets treated with strawberry extract. In vivo strawberry extracts injected intraperitoneally in mice	In vitro platelets from 6 healthy volunteers In vivo C57BL/6 mice 12-16-week old with photochemical induced thrombosis	Reduced platelet aggregation in vitro and thrombus formation in vivo	Strawberry extract inhibits thrombus formation and targets platelet activation responses
Moazen et al. 2013 [68]	Daily consumption of 2 cups freeze-dried strawberries (FDS) containing 154 mg anthocyanins for 6 weeks	23 females with T2D average age 51.5 years	Reduced C-reactive protein (CRP), lipid peroxidation, and HbA1c and increased total antioxidant status	Strawberry improved glycemic control, and reduced inflammation and oxidative stress in T2D patients
Basu et al. 2010 [69]	Consumption of 4 cups of FDS beverage daily for 8 weeks	27 subjects (2 male 25 female, mean age 47) with metabolic syndrome	Decreased total and LDL-cholesterol, and vascular cell adhesion molecule 1 (VCAM-1) levels	Strawberry improved CVD risk factors including dyslipidemia and VCAM-1 level
An et al. 2016 [70]	Consumption of *Rubus occidentalis* extract at low (900 mg/day) or high dose (1800 mg/day) for 12 weeks	44 prediabetic patients (31 female, 13 male, mean age 59 years)	High dose reduced glucose levels, while MCP-1 and oxidized LDL were reduced in a dose dependent manner	*Rubus occidentalis* improved glycemic control and vascular inflammation in prediabetic patients
Suh et al. 2011 [71]	Consumption of atherogenic diet with a daily dose of cranberry juice varieties for 12 weeks	10 weanling male Syrian golden hamsters	Reduced superoxide, plasma triglycerides, and increased GPx. Cardinal juice, decreased LDL and increased high-density lipoprotein (HDL) level	Consuming various cranberry juices can reduce atherosclerosis by increasing antioxidant capacity and improving lipid profile
Pala et al. 2018 [72]	Daily consumption of 200 g of acai pulp for 4 weeks	40 healthy women average age 24 years	Acai increased total antioxidants and cholesterol in HDL. Decreased ROS and oxidized LDL levels	Acai fruit reduced oxidative stress and improved cholesterol transport that could reduce atherosclerosis

**Table 2:** Effect of vegetables and olive oil in risk factor of CVD.

Author, Year	Treatment	Model	Result	Conclusion
Tsang et al. 2018 [73]	Consumption of cooked purple potato (200 g/day containing 288 mg anthocyanins) for 14 days	14 healthy, non-smoking male and female adults age 22–55 years	Decreased pulse wave velocity compared to white potato	Purple majesty potato may reduce arterial stiffness, an independent indicator of CVD
Mattioli et al. 2018 [74]	Self-reported fruit and vegetable intake through FFQ Ankle-brachial index (ABI) was used to assess preclinical atherosclerosis	237 women with hypertension age 45–54 years	Fruit and vegetable intake was associated with reduced ABI, while vegetable intake was associated with reduced risk of periphery artery disease	Fruit and vegetable consumption is associated with a lower risk of atherosclerosis in pre-menopausal women
Lahoz et al. 2010 [75]	Adherence to a Mediterranean diet based on the Mediterranean diet adherence screener	1411 subjects (43% male, mean age 61 years)	Inverse relationship between Mediterranean diet and CRP levels	Mediterranean diet, rich in fruits and vegetables, decreases atherosclerotic risk factors
Juan et al. 2017 [76]	Fruit and vegetable intake reported by FFQ in in-person interviews	Case study of 2158 Chinese subjects (918 families) testing association of *PON1 rs662* polymorphism with risk of stroke	Individuals with *PON1 rs662* gene reduced the risk of stroke and atherosclerosis with fruit intake	Fruits and vegetables reduce risk of stroke in individuals with *PON1 rs662* polymorphism
Woo et al. 2018 [77]	Dietary estimation through self-reported FFQ	Cross sectional analysis of 4000 men and women aged 65 years and over in Hong Kong, China	High score on fruit and vegetable intake or Mediterranean diet decreased ABI	Fruit and vegetable consumption is associated with better cardiovascular health
Carnevale et al. 2018 [78]	0.2 mg/mL extra virgin olive oil (EVOO) with or without 500 U/mL catalase and stimula-ted for 10 min with 0.5 mM arachidonic acid	Blood platelets taken from 5 healthy subjects (3 male, 2 female, mean age 39.8 years)	Both catalase and EVOOs reduced the activation of Nox2 and H_2_O_2_ production	EVOO reduced oxidative stress by decreasing H_2_O_2_ levels and Nox2 activity
Farras et al. 2018 [79]	3-week consumption of 25 mL/day virgin olive oil (VOO), plus its own polyphenols (FVOO), or plus thyme polyphenols (FVOOT)	33 hypercholesteremic individuals	FVOO and FVOOT increased HDL, while FVOOT increased α-tocopherol	Olive oil enriched in polyphenols increases HDL levels and antioxidant capacity
